# Evidence-based interconversion of the Glasgow Outcome and modified Rankin scales: pitfalls and best practices

**DOI:** 10.1016/j.jstrokecerebrovasdis.2022.106845

**Published:** 2022-10-26

**Authors:** Ben Gaastra, Dianxu Ren, Sheila Alexander, Issam A Awad, Spiros Blackburn, Sylvain Doré, Dan Hanley, Paul Nyquist, Diederik Bulters, Ian Galea

**Affiliations:** a Clinical Neurosciences, Clinical and Experimental Sciences, Faculty of Medicine, University of Southampton, Southampton SO17 6YD, UK; b Department of Neurosurgery, Wessex Neurological Centre, University Hospital Southampton NHS Foundation Trust, Southampton SO16 6YD, UK; c School of Nursing, University of Pittsburgh, Pittsburgh, PA, United States; d University of Chicago, Chicago, IL, United States; e University of Texas Houston Health Science Center, Houston, TX, United States; f Departments of Anesthesiology, Neurology, Psychiatry, Pharmaceutics, and Neuroscience, College of Medicine, Center for Translational Research in Neurodegenerative Disease, McKnight Brain Institute, University of Florida, Gainesville, FL, United States; g Johns Hopkins School of Medicine, Baltimore, MD, United States.

**Keywords:** Stroke, Outcome, Modified Rankin scale, Glasgow outcome scale

## Abstract

**Objective:**

The aim of this study was to provide the evidence base to guide interconversion of the modified Rankin Scale (mRS) and Glasgow Outcome Scale (GOS) in neurological research.

**Methods:**

A retrospective analysis of paired mRS and GOS recordings was conducted using datasets with the following selection criteria: (1) patients had haemorrhagic stroke, (2) simultaneous mRS and GOS measurements were available, and (3) data sharing was possible. The relationship between mRS and GOS was assessed using correlation analysis. The optimum dichotomisation thresholds for agreement between the mRS and GOS were identified using Cohen’s kappa coefficient. Two-way conversion tables between mRS and GOS were developed based on the highest agreement between scores. Finally, to identify which direction of conversion (mRS to GOS or vice versa) was better, the Kolmogorov-Smirnov D statistic was calculated.

**Results:**

Using 3474 paired recordings the mRS and GOS were shown to be highly correlated (*ρ* = 0.90, *p* < 0.0001). The greatest agreement between the two scoring systems occurred when mRS=0–2 and GOS=4–5 was used to define good outcome (κ=0.83, 95% confidence interval: 0.81–0.85). Converting from mRS to GOS was better than the reverse direction as evidenced by a lower Kolmogorov-Smirnov statistic (D=0.054 compared to D=0.157).

**Conclusions:**

This study demonstrates that the mRS and GOS are highly correlated, establishes the optimum dichotomisation threshold for agreement, provides a method for interconversion and shows that mRS to GOS conversion is superior to the reverse direction if a choice is available.

## Introduction

**Fig. 1.**
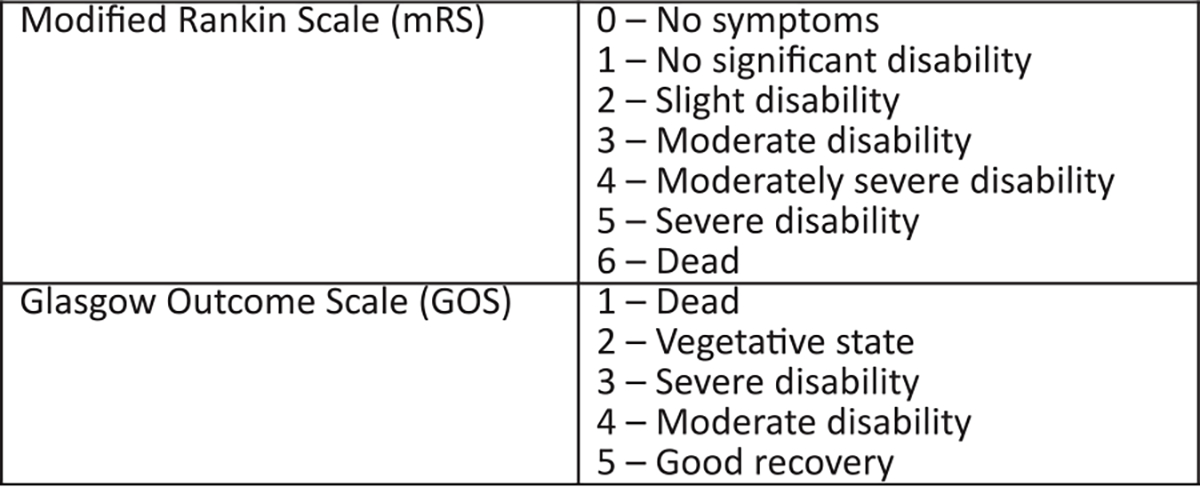
The modified Rankin Scale (mRS) and Glasgow Outcome Scale (GOS).

The modified Rankin (mRS)^1,2^ and Glasgow Outcome Scales (GOS)^3^ are commonly used clinical outcome assessment tools in neurological research, especially in stroke. Both scales grade individuals according to functional recovery with the mRS ranging from 0 (no symptoms) to 6 (death) and the GOS from 1 (death) to 5 (good recovery) (Fig. 1).

Haemorrhagic stroke, including intracerebral haemorrhage (ICH) and aneurysmal subarachnoid haemorrhage (aSAH), only represents a small fraction of all strokes (15%). Consequently, observational studies of outcome following haemorrhagic stroke often rely on pooling data from multiple centres to increase sample size. As individual centres may adopt either the mRS or GOS, but rarely both, researchers commonly encounter the need to interconvert the two scales to allow pooled analysis. This interconversion is typically conducted by dichotomising the individual mRS and GOS scores into “good” and “poor” outcome and then pooling.

A single National Institute of Neurological Disorders and Stroke (NINDS) workshop reported strong agreement between the mRS and GOS in the context of ischaemic stroke^4^ and this is also likely to be the case for haemorrhagic stroke. There is, however, no evidence base in the literature guiding interconversion of the two scales. Specifically, the precise dichotomisation thresholds defining “good/poor” outcome in the mRS and GOS, which result in the best agreement between the two scales, has not been established. This information is essential to facilitate rigorous retrospective meta-analyses of individual patient level data from multiple studies employing both scales. We set out to address this unmet need, taking advantage of available datasets and adopting a rigorous statistical approach. Specifically in the context of haemorrhagic stroke our aims were to: (1) assess the correlation between the mRS and GOS, (2) identify which dichotomisation thresholds for “good/poor” outcome result in the best agreement between the two scales, and (3) develop the evidence base to enable conversion between the two scales.

## Methods

This study used published data with institutional approval (ERGO 31523.A1). Patients with haemorrhagic stroke were identified from three international multicentre studies: (1) a retrospective observational analysis of outcome following aSAH^5^, (2) CLEAR III, a randomised controlled trial of thrombolytic therapy for intraventricular haemorrhage (IVH)^6^, and (3) MISTIE III, a randomised controlled trial of minimally invasive surgery with thrombolysis for ICH.^7^ All paired mRS and GOS values, collected at the same timepoint, between 1 and 12 months were eligible for inclusion, meaning individuals could contribute multiple paired values.

Spearman’s rank correlation coefficient, rho (ρ), was used to assess the relationship between scales. Cohen’s kappa coefficient was used to assess which mRS and GOS dichotomisation thresholds defining “good/poor” outcome showed the best agreement. A higher kappa value indicates stronger agreement. For this analysis, death and severe disability (GOS=1–3 and mRS=4–6) were excluded from the definition of “good” outcome because these are universally regarded as “poor” outcome.

To develop a conversion table between the mRS and GOS, individual scores were cross-tabulated, and two-way conversions assigned based on the highest agreement between scores. At this level of agreement, we computed the probability that an individual’s true score was the same as the converted score. Finally, to identify which direction of conversion (mRS to GOS or vice versa) was better, the Kolmogorov-Smirnov statistic, describing the maximum vertical distance between empirical cumulative distribution functions of directly acquired and converted scores, was calculated. Kuiper, Cramer-von Mises and Anderson-Darling statistical tests were also performed as sensitivity analyses to support the findings. A smaller statistic reflects more similar distributions for all of these tests.

To test the interconversion methods proposed in this study we used published data from the INTERACT2 trial.^8^ INTERACT2 was a randomised controlled trial designed to assess the benefit of intensive blood pressure lowering compared to guideline-recommended treatment following spontaneous ICH. The primary outcome was the proportion of participants with poor outcome, defined as mRS=3–6, at 90 days. To assess performance of the proposed mRS/GOS interconversion methods we converted the mRS scores from INTERACT2 to GOS to see if interconversion would influence the primary study outcome.

Statistical analyses were performed in SAS (SAS Institute) and R (version 4.1.2, R Foundation for Statistical Computing). Data are available from the authors subject to institutional agreements and ethical approvals.

## Results

A total of 1495 individuals with 3474 paired mRS and GOS recordings between 1 and 12 months post haemorrhagic stroke were identified for inclusion (see Table 1 for demographics, sample size and number of paired mRS/GOS values in each study).

The mRS and GOS were strongly correlated (*ρ* = 0.90, *p* < 0.001). When analysed individually, all three datasets showed the same direction and significance of results (aSAH: *ρ* = −0.88, p < 0.001; IVH: *ρ* = −0.84, *p* < 0.001; ICH: *ρ* = −0.82, *p* < 0.001).

The kappa statistic, commonly used to assess agreement on a nominal scale, was employed to establish the optimum dichotomisation threshold between the mRS and GOS. The greatest agreement (highest kappa) was seen when a dichotomisation threshold of mRS=0–2 and GOS=4–5 was used to define good outcome (κ=0.83, Table 2).

In order to provide the research community with a tool to convert from one scale to the other, we cross-tabulated the mRS and GOS to find the highest levels of agreement, and based on this, we provide two-way conversion tables in Table 3. The probability that an individual’s true score was the same as their converted score is also shown.

**Fig. 2.**
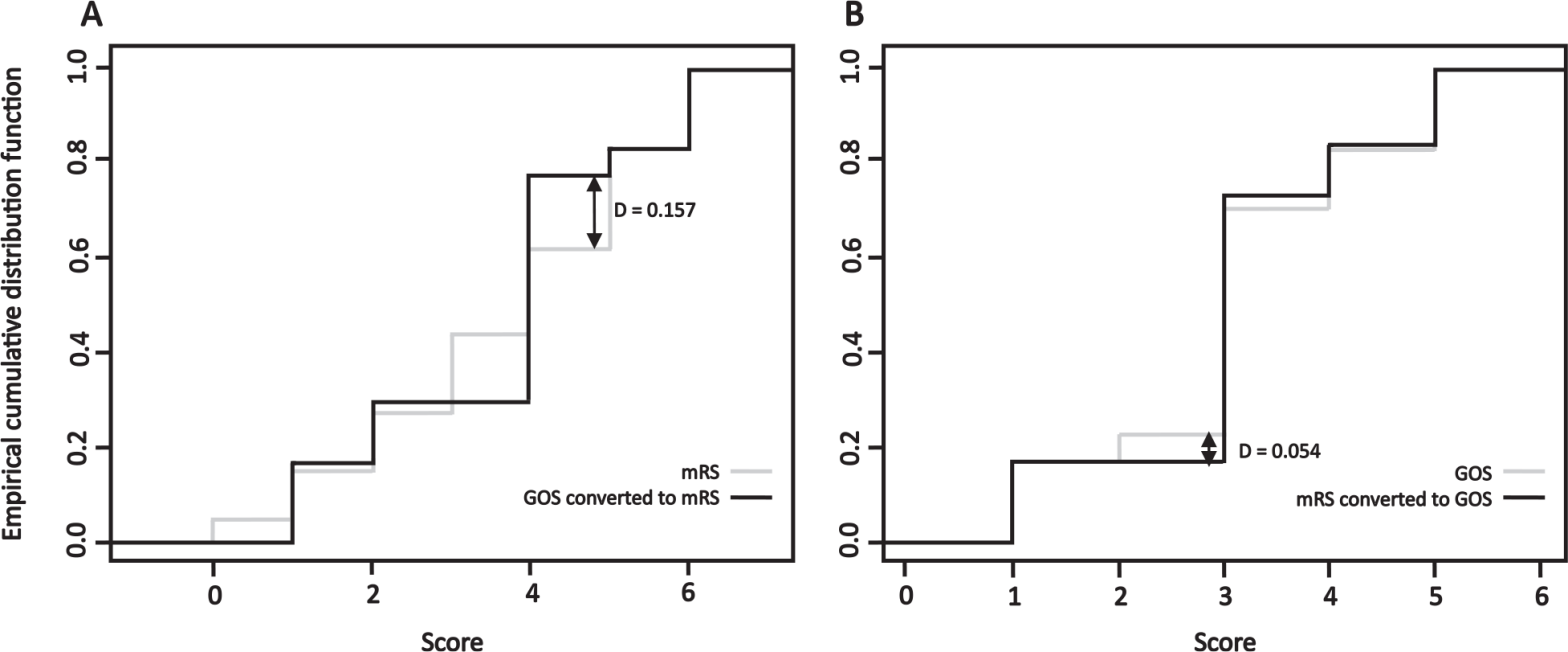
Empirical cumulative distribution functions comparing A: mRS directly acquired from patients and GOS converted to mRS; and B: GOS directly acquired from patients and mRS converted to GOS. The arrows signify the maximum vertical distance between the two empirical distribution functions with a smaller value representing more similar data distributions (Kolmogorov-Smirnov statistic (D)).

When both the mRS and GOS are available from studies within an individual patient level data meta-analysis, it is important to know whether one conversion direction is superior to the other. To this end we used the D statistic in a two sample Kolmogorov-Smirnov test, comparing the empirical cumulative distribution functions of the scale before and after conversion. Converting from mRS to GOS was better than the reverse direction since the empirical cumulative distribution functions of directly acquired and converted scores were closer to each other in the mRS to GOS direction (D=0.054 compared to D=0.157, Table 4, Fig. 2). The direction of conversion is further supported by similar results using the Kuiper, Cramer-von Mises and Anderson Darling tests (Table 4).

As an exemplar, we converted the directly acquired mRS scores from the INTERACT2 study to GOS using the proposed conversion methodology (Table 3), and results are presented in Table 5. The primary outcome in the INTERACT2 study was based on the mRS, with poor outcome defined as mRS=3–6. Using the dichotomisation threshold agreements from Table 2 this definition of poor outcome equates to GOS=1–3. When the primary outcome of the INTERACT2 study was re-analysed using the converted GOS scores, the proportion of participants with poor outcome was identical to that of the original analysis using directly acquired mRS scores (Table 5).

## Discussion

In this study we show that mRS and GOS are highly correlated following haemorrhagic stroke and provide the evidence base to allow interconversion of the two scales. Moreover, we test the interconversion methodology in an external dataset, with excellent results.

The mRS was specifically developed to assess outcome following stroke, although predominantly in ischaemic rather than haemorrhage stroke.^2^ GOS, on the other hand, was designed in the context of head injury^3^ and its use in stroke has been questioned.^9^ The GOS, however, remains a commonly used outcome score, especially in haemorrhagic stroke, emphasising the importance of understanding its relationship to the mRS. While prospective studies should collect the same consistent outcome measure, retrospective individual patient level studies may require the combined use of existing mRS and GOS datasets, for which this study provides the appropriate evidence base and methodology. As observed in a single publication of ischaemic stroke^4^ the mRS and GOS are also highly correlated in haemorrhagic stroke, both overall and within individual haemorrhage types.

No previous studies have assessed the optimum dichotomisation threshold for agreement between mRS and GOS. In this study the kappa statistic identified that the best agreement occurred using a dichotomisation threshold of GOS=4–5 and mRS=0–2 to define “good” outcome (κ=0.83, 95% confidence interval: 0.81–0.85, Table 2), supporting the use of this threshold when pooling dichotomised datasets. This dichotomisation includes GOS=4 (moderate disability, Fig. 1), which is not universally considered as a “good” outcome. If moderate disability is excluded from the definition of “good” outcome then the best agreement occurs when mRS=0–1 and GOS=5 are used to define “good” outcome (κ=0.81, 95% confidence interval: 0.79–0.84, Table 2); this threshold excludes mRS=2 (slight disability, Fig. 1) from the definition of “good” outcome. As the kappa statistic 95% confidence intervals overlap for these two dichotomisation thresholds, it is not immediately obvious that one is superior to the other and the decision should be guided by study context.

Defining “good” outcome as mRS=0–1 may be preferable when one considers Table 3, which provides the score-by-score interconversion between the mRS and GOS (with the probability that an individual’s true score was the same as their converted score). When converting from mRS to GOS, a score of GOS=2 had a low probability (see Table 3 and legend); this was expected as there is no equivalent of GOS=2 (vegetative state) in the mRS. When converting from GOS to mRS, a score of mRS=3 also had a low probability (see Table 3 and legend). Although some incongruence is expected when converting a 5-point (GOS) to a 7-point scale (mRS), the low probability of conversion to GOS=2 and mRS=3 does limit the use of the conversion tables. It, therefore, follows that dichotomization into “good” and “poor” outcome is preferable to using the conversion tables. Moreover, dichotomisation of scores avoiding mRS=3 on the boundary between “good” outcome and “poor” outcome, i.e. defining “good” outcome as mRS=0–1, may offer the most reliable way of merging mRS and GOS data.

In pooled mRS and GOS datasets, when a decision is made to convert scores rather than dichotomize into “good” and “poor” outcome, and a choice of conversion direction is available, mRS to GOS conversion is superior to the reverse direction. This is likely because the GOS has fewer levels than the mRS reducing the spread of distribution. It should be noted that converting from the seven-point mRS to the five-point GOS may reduce the sensitivity of outcome assessment, for example the mRS has five levels covering what is conventionally considered to be good to moderate outcome whereas the GOS only has two such levels.

A major strength of this study is the large sample size drawn from multiple international cohorts, including different types of haemorrhagic strokes and study designs (observational and randomised controlled trials), enhancing generalizability. We also demonstrate in an external cohort that the proposed dichotomisation threshold and method of score interconversion does not alter the results of the INTERACT2 randomised controlled trial. This supports the application of these interconversion methods to external datasets.

Prior to this study there was no evidence base to support the interconversion of mRS and GOS in neurological disease. This study adds to the literature by providing practical methodological approaches to interconvert mRS and GOS. This will allow researchers to pool multiple studies using these different outcome measures to generate large sample sizes. Ultimately, analyses of large datasets will further our understanding of neurological disease and impact clinical practice, for example, by providing valuable prognostic information, improving outcome prediction and advancing our understanding of the pathophysiological mechanisms involved.

### Limitations

A number of limitations merit discussion. This study focussed on the GOS and not its 8-point extended version (GOS-E)^10^ but this was purposeful, since the GOS-E can be easily converted to the GOS, but not vice versa. Another limitation is that the dataset did not include other neurological conditions. While caution may be required when translating the results from haemorrhagic stroke to other conditions, one would expect the basic principles of disability assessment by the mRS and GOS, and the mathematical relationship between the scores, to be agnostic of the pathological process leading to neurological disability. When it is necessary to follow disability with time in the same patient, it is important to ensure when possible that outcome is measured with the same scale, and ideally by the same rater to minimize the effects of inter-rater reliability.^11,12^

### Future directions

The results of this study are based on individuals with haemorrhagic stroke and tested in a randomised controlled trial in ICH. Although it is likely that the results are translatable to other neurological conditions future studies are required to validate the findings in different cohorts. In addition to GOS and mRS other disease-specific outcome scales are being increasingly used in research, for example the Subarachnoid Haemorrhage Outcome Tool (SAHOT).^13^ Future work is required to assess how these scales correlate with mRS and GOS to maximise the number of studies that can be included in pooled analyses.

## Conclusion

In the context of haemorrhagic stroke, this study demonstrates that the mRS and GOS are highly correlated, establishes the optimum dichotomisation threshold for agreement, provides a method for interconversion and shows that mRS to GOS conversion is superior to the reverse direction if a choice is available.

## Figures and Tables

**Table 1.** Demographics of patients included in the study. Two individuals were excluded from the study as they were coded as dead according to the GOS but as severe disability on the mRS. A sensitivity analysis including these individuals made no difference to the direction and significance of the results. SD: standard deviation, IPLD: individual patient level data.

Study	Haptoglobin IPLD^3^	CLEAR III^4^	MISTIE III^5^

Stroke type	Aneurysmal subarachnoid haemorrhage	Intraventricular haemorrhage	Intracerebral haemorrhage
Sample size	496	499	500
Number of paired mRS and GOS values	1030	968	1476
Age, mean (SD)	54.6 (12.7)	58.6 (11.2)	61.2 (12.4)
Sex, n (%)			
Male	140 (28)	277 (56)	307 (61)
Female	352 (71)	222 (44)	193 (39)
Missing	4 (1)	-	-

**Table 2.** Kappa statistics (95% confidence interval) and number of samples included in analysis (n, %) of agreement between dichotomised modified Rankin Scale (mRS) and Glasgow Outcome Scale (GOS) at different thresholds to separate “good” and “poor” outcome. Higher kappa values indicate better agreement. Death and severe disability (GOS 1–3 and mRS 4–6) were excluded from the definition of “good” outcome because these are universally regarded as “poor” outcome.

		GOS	
	
		5	4–5

	0	0.41 (0.37, 0.45)	0.24 (0.21, 0.27)
		*n* = 3041, 87.5%	*n* = 2627, 75.6%
	0–1	0.81 (0.79, 0.84)	0.62 (0.59, 0.65)
mRS		*n* = 3291, 94.7%	*n* = 2995, 86.2%
	0–2	0.70 (0.67, 0.73)	0.83 (0.81, 0.85)
		*n* = 3110, 89.5%	*n* = 3230, 93.0%
	0–3	0.42 (0.40, 0.45)	0.67 (0.65, 0.70)
		*n* = 2552, 73.5%	*n* = 2932, 84.4%

**Table 3.** Conversion table between mRS and GOS. To develop the conversion table, directly acquired mRS and GOS scores were cross-tabulated and the best conversion is displayed, based on the highest probability of agreement between the two scores. When mRS is converted to GOS the best performing conversion does not include GOS=2, since the probability of agreement for this category was lower (P [mRS 5 → GOS 2] = 0.2558). When GOS is converted to mRS, the best performing conversion does not include mRS=3, since the probability of agreement for this category was lower (P [GOS 4 → mRS 3] = 0.3023).

mRS	Converted GOS	Probability of agreement

0	5	0.9581
1	5	0.8272
2	4	0.5517
3	3	0.7578
4	3	0.9612
5	3	0.7401
6	1	1.0000

GOS	Converted mRS	Probability of agreement

1	6	1.0000
2	5	0.9947
3	4	0.3591
4	2	0.4837
5	1	0.5197

**Table 4.** The Kolmogorov-Smirnov statistic D, or maximum vertical distance between empirical cumulative distribution functions of directly acquired and converted scores, was computed. Conversions were performed using the data presented in Table 3. As indicated by a smaller D statistic, the direction mRS → GOS was better than GOS → mRS. Kuiper, Cramer-von Mises and Anderson-Darling test statistics are also shown as sensitivity analyses to demonstrate robustness of findings; a smaller statistic reflects more similar distributions.

Distribution comparison	Kolmogorov-Smirnov statistic (D)	Kuiper test statistic	Cramer-von Mises test statistic	Anderson-Darling test statistic

Directly acquired mRS & GOS converted to mRS	0.157	0.298	0.048	4.593 × 10^−5^
Directly acquired GOS & mRS converted to GOS	0.054	0.080	0.004	3.239 × 10^−6^

**Table 5.** The directly acquired mRS and converted GOS for the two arms of the INTERACT2 study including the proportion of individuals with poor outcome (the primary INTERACT2 study outcome). The scores were converted using the conversion tables in Table 3.

	Intensive blood-pressure lowering (*n*=1382)	Guideline-recommended blood pressure lowering (*n*=1412)

Directly acquired mRS		
0	112 (8.1%)	107 (7.6%)
1	292 (21.1%)	254 (18.0%)
2	259 (18.7%)	266 (18.8%)
3	220 (15.9%)	234 (16.6%)
4	250 (18.1%)	268 (19.0%)
5	83 (6.0%)	113 (8.0%)
6	166 (12.0%)	170 (12.0%)
Primary outcome: mRS=3–6	719 (52.0%)	785 (55.6%)
Converted GOS		
1	166 (12.0%)	170 (12.0%)
2	-	-
3	553 (40.0%)	615 (43.6%)
4	259 (18.7%)	266 (18.8%)
5	404 (29.2%)	361 (25.6%)
Converted primary outcome: GOS=1–3	719 (52.0%)	785 (55.6%)

## Abbreviations:

mRS: modified Rankin Scale
GOS: Glasgow Outcome Scale
ICH: Intracerebral Haemorrhage
aSAH: aneurysmal Subarachnoid Haemorrhage
IVH: Intraventricular Haemorrhage
NINDS: National Institute of Neurological Disorders and Stroke
SAHOT: Subarachnoid Haemorrhage Outcome Tool
